# Hydrogen Isotopes as a Sentinel of Biological Invasion by the Japanese Beetle, *Popillia japonica* (Newman)

**DOI:** 10.1371/journal.pone.0149599

**Published:** 2016-03-09

**Authors:** Bruce A. Hungate, Diana N. Kearns, Kiona Ogle, Melanie Caron, Jane C. Marks, Helmuth W. Rogg

**Affiliations:** 1 Center for Ecosystem Science and Society, Colorado Plateau Stable Isotope Laboratory, and Department of Biological Sciences, Northern Arizona University, Flagstaff, AZ, 86011, United States of America; 2 Oregon Department of Agriculture, Plant Division, Salem, OR, 97301, United States of America; 3 Informatics and Computing Program, Northern Arizona University, Flagstaff, AZ, 86011, United States of America; Universidad Nacional Autonoma de Mexico, MEXICO

## Abstract

Invasive species alter ecosystems, threaten native and endangered species, and have negative economic impacts. Knowing where invading individuals are from and when they arrive to a new site can guide management. Here, we evaluated how well the stable hydrogen isotope composition (δ^2^H) records the recent origin and time since arrival of specimens of the invasive Japanese beetle (*Popillia japonica* Newman) captured near the Portland International Airport (Oregon, U.S.A.). The δ^2^H of Japanese beetle specimens collected from sites across the contiguous U.S.A. reflected the δ^2^H of local precipitation, a relationship similar to that documented for other organisms, and one confirming the utility of δ^2^H as a geographic fingerprint. Within weeks after experimental relocation to a new isotopic environment, the δ^2^H of beetles changed linearly with time, demonstrating the potential for δ^2^H to also mark the timing of arrival to a new location. We used a hierarchical Bayesian model to estimate the recent geographical origin and timing of arrival of each specimen based on its δ^2^H value. The geographic resolution was broad, with values consistent with multiple regions of origin in the eastern U.S.A., slightly favoring the southeastern U.S.A. as the more likely source. Beetles trapped from 2007–2010 had arrived 30 or more days prior to trapping, whereas the median time since arrival declined to 3–7 days for beetles trapped from 2012–2014. This reduction in the time between arrival and trapping at the Portland International Airport supports the efficacy of trapping and spraying to prevent establishment. More generally, our analysis shows how stable isotopes can serve as sentinels of biological invasions, verifying the efficacy of control measures, or, alternatively, indicating when those measures show signs of failure.

## Introduction

In regions where a particular invasive species is not known to be established, capturing an individual of that species raises a key question: is it from an established population, or is it a new arrival [1,2]? The answer is important, because management actions can focus on documenting the extent of the invasion and eradication if the capture is known to come from an established population [1]. On the other hand, if an individual is a recent arrival from a source population elsewhere, monitoring for new arrivals and improved containment at the source populations may be more effective in reducing the probability that the invasion establishes. In other words, managing invasions improves when the status of the invasion is well understood [3,4]. Yet, for species known to threaten invasion, current techniques often do not make it possible to distinguish between new arrivals and members of establishing populations. The goal of this work was to test whether stable isotopes of hydrogen can serve as sentinels of biological invasion by the Japanese beetle (*Popillia japonica* Newman), distinguishing new arrivals from established residents.

**Fig 1:**
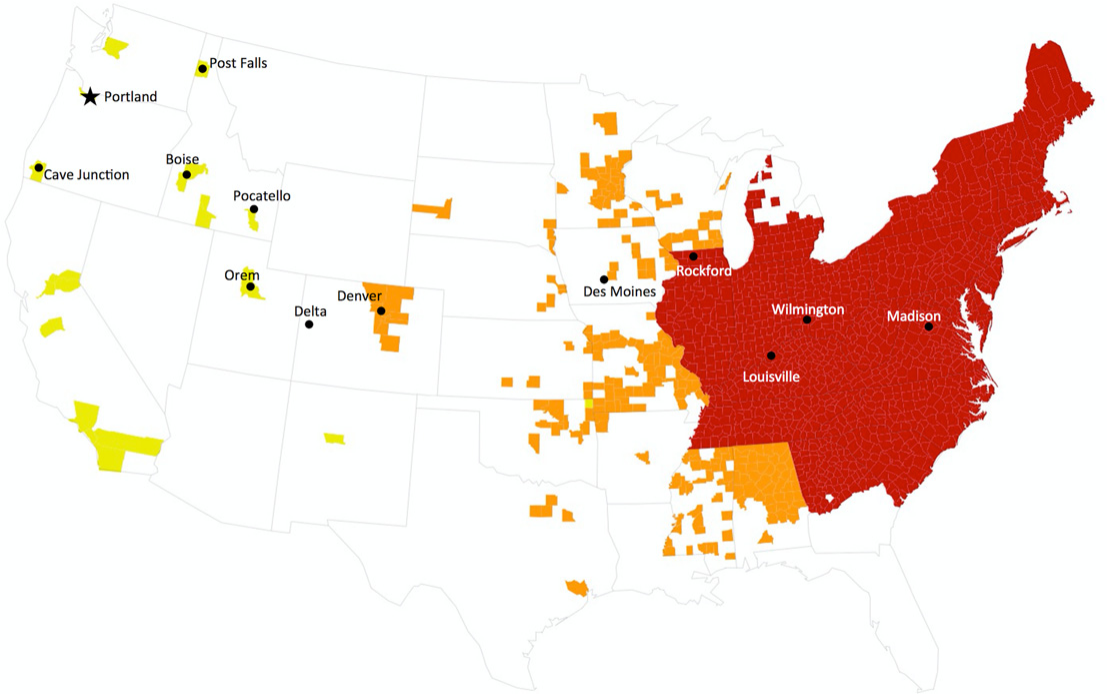
Map of continental US showing the status of the Japanese beetle invasion, by county: well established (red), establishing (orange), occurrences reported (yellow), and no occurrences known (white). Black circles indicate sites of beetle collections used to develop the relationship between beetle and precipitation δ^2^H values, and the star indicates the study site at the Portland International Airport. Data compiled from refs 15–19, as described in the Methods (***Potential Sources of Origin)*.**

The Japanese beetle was first found in the continental U.S.A. in southern New Jersey in 1916 [5], and it is now established throughout the eastern U.S.A. (Fig 1), threatening western states. This voracious beetle is damaging because it feeds on plant roots, foliage, and flowers, and is a generalist herbivore with over 300 plant species, including grasses used for turf [6], various crops [7], and horticultural plants [8]. Grubs (the immature form) overwinter until spring and then feed on roots until they pupate in the soil for two to three weeks. When they emerge as adults, they mate and continue to feed on plant foliage and flowers for up to 60 days, after which they lay eggs in the soil that hatch that same year; grubs continue to feed in the soil until fall when they overwinter [5]. Damage caused by Japanese beetles is likely to increase with rising CO_2_ and global warming because of increased foliage consumption rate [9], and they also exacerbate damage caused by other herbivores [10].

Preventing expansion of this pest into the western U.S.A. is a high priority. Extensive measures are used in airports in the eastern U.S.A. to prevent individual beetles from entering cargo planes, including inspections and exclusion devices around doors, hatches, and other openings prior to departure, as well as pesticide applications at sites of capture [11]. Despite these measures, Japanese beetles have been caught at the Portland International Airport in Oregon since 1992, and every year since 2000. Each year, ground pesticide treatments that target the immature soil-dwelling beetles are applied in May. Foliar sprays targeting adult beetles were conducted in July and August 2002–2014. Other western states (Washington, California, Idaho, Utah, and Colorado) have also intercepted this quarantine pest at airports, in imported nursery stock, and in urban areas in recent years [11–17]. The distinction between new arrivals or members of establishing populations becomes more difficult in areas around airports that receive many flights harboring potential invaders. Multiple individual captures could indicate strong propagule pressure, or alternatively, the growth of a nascent population, both of concern for invasive species, but with different management implications [18].

Variation in the isotope composition of animals reflects their natural history–what they eat, what they drink, and where they travel–providing a forensic tool for animal ecology [19–21]. Hydrogen in animal tissue comes from ingested hydrogen, both as water and organic matter. Plant tissue hydrogen isotope composition (δ^2^H) reflects that of local precipitation, offset by isotope effects associated with photosynthesis and evaporation [22]. Thus, the hydrogen isotope signature in animal tissue should also reflect the isotopic composition of local precipitation [23,24], which displays broad geographic variation [25,26]. Across the U.S.A., regional variation in δ^2^H in precipitation is strong, with high values in the southeastern U.S.A., and decreasing systematically to the northwest.

Chitin and keratin, key substances in insect structure (in the formation of the exoskeleton and wings) are biosynthesized with the incorporation of hydrogen isotopes from diet and water in the environment [21]. Once chitin and keratin are synthesized, the majority of hydrogen atoms are non-exchangeable with ambient water vapor [27]. For this reason, hydrogen isotope ratios in chitinous and keratinous tissues have proven valuable for determining the natal origins of organisms [19,21,24,28–32], including of invasive species [33].

Other insect tissues are metabolically active and there is capacity for turnover of isotope signatures in new environments [34]. Isotope signatures in these tissues could determine whether an individual is a recent arrival, reflecting the isotopic signature of its region of origin, or whether it represents an established population, isotopically equilibrated with the new environment [28,35]. This could also provide useful information about invasive species.

Here, we analyzed δ^2^H signatures of Japanese beetles of known origin across the U.S.A. as a foundation for examining the continental-scale variation in δ^2^H of Japanese beetles. We tested experimentally how beetle δ^2^H shifts over a 5-week period when exposed to a new isotopic environment, and incorporated the findings into a hierarchical Bayesian model that describes the timing and origins of Japanese beetles caught at the Portland International Airport. This work assesses the utility of δ^2^H signatures of invasive organisms as sentinels of the invasion status, and as a potential foundation for making decisions about control and eradication efforts.

## Materials and Methods

### Hydrogen Isotope Composition of Field-Collected Japanese Beetles

Voucher specimens of Japanese beetles collected from field traps were sent from Illinois, Iowa, Kentucky, and Ohio (n = 10 for each location), and from multiple sites in Colorado (n = 15), Utah (n = 5), and Idaho (n = 11), a total of 71 samples from known populations. Specimens from Iowa, Kentucky, Ohio, and Illinois were provided in fall, 2010 by MTA Horticultural Consultants in Middletown, KY. The Utah samples were sent in November 2012 by the Utah Department of Agriculture, and the Colorado samples in 2012 by the Colorado Department of Agriculture. The samples from Idaho were collected between July and August 2012, and were provided by the Idaho Department of Agriculture. Specimens were kept frozen at -10°C in glass vials to reduce isotopic exchange with ambient water vapor [36] and were shipped overnight to the Colorado Plateau Stable Isotope Laboratory (CPSIL, www.isotope.nau.edu), where they were dried before analysis. No permits or approvals were required for the field sampling, because the Japanese beetle is an invasive organism in all of the sites sampled, not requiring permission for removal. To compare δ^2^H values of beetles with δ^2^H of precipitation, we used the on-line isotopes in precipitation calculator (OPIC, [26,37]) to estimate the annual mean and standard error of δ^2^H in precipitation for each site where beetle specimens were collected. For the hierarchical Bayesian modeling, monthly values for each site were also estimated from OPIC and error terms estimated as described below.

Japanese beetles were collected from the Portland International Airport (PDX) and in several sites within 10 miles of the airport from 2006–2014. The beetles were collected in Japanese beetle traps placed in the field, checked on average every 3–4 weeks in earlier years (2006–2011) and every 3 days by camera in later years (2012–2015). Beetles were removed as soon as they were detected in the traps. Traps consist of a metal funnel top that has crossed panels that lead down to a collection can on the bottom. The upper portion of the trap is baited with a scented lure, a Trece Pherocon dual lure for Japanese beetle, which has two components, a floral attractant and a pheromone attractant. Based on observations of traps equipped with cameras installed since 2012, beetles typically die in the traps within 24 hours, minimizing isotope exchange with the surrounding environment due to metabolism. Furthermore, there was no relationship between the number of days between setting the trap and finding a dead beetle inside (r = 0.16), indicating that time in the trap had little influence on the isotope value of the beetle. Specimens recovered from traps were placed in glass vials and stored at -10^0^ C.

### Isotope Turnover Experiment

Approximately 200 beetles were shipped overnight on 16 July 2013 from the Virginia Department of Agriculture (Madison, Virginia, U.S.A.) for an experiment designed to determine whether beetle δ^2^H changes after relocation to a region with distinctly different δ^2^H in precipitation and plant material, and, if a change occurs, at what rate. These beetles were housed in a quarantine facility starting 17 July 2013. The facility was established in Salem, Oregon, U.S.A. in Spring 2013; the facility met all requirements for containment by the U.S. Department of Agriculture Animal and Plant Health Inspection Service Plant Protection and Quarantine agency (USDA APHIS PPQ). This agency granted a permit to move live plant pests. Within the facility, beetles were placed in a terracosm with a screen top in the quarantine area on July 17, 2013, and were provided with local rose plants (δ^2^H = -121‰ for leaf tissue) and water from a local well (δ^2^H = -66‰) during a 5-week experiment. The plant δ^2^H value is consistent with that expected for local vegetation, though the water δ^2^H was lower than that of local precipitation (δ^2^H = -88‰, CI 6‰, from OPIC [37]). Thus, to the extent ingestion of water influences beetle δ^2^H, our experiment likely underestimated isotope turnover.

Ten beetles were immediately frozen to determine initial δ^2^H. Each week over a 5-week period, 10 beetles were harvested from the terracosm. Specimens were frozen immediately and then dried. Individual beetles collected in the first and last week’s samples were dissected to separate the elytra, membranous wings, and bodies, and each of these body parts was analyzed separately for δ^2^H. This tested the idea that elytra and wings, high in chitin and keratin concentration, are less prone to changes in δ^2^H in response to a change in the δ^2^H of water and food sources, because non-exchangeable hydrogen is thought to be stable in chitin and keratin once they are synthesized [27].

### Isotope Analyses

All samples were analyzed for δ^2^H at CPSIL at Northern Arizona University. All field specimens were analyzed using the entire beetle; some samples from the isotope turnover experiment were divided into membranous wings, elytra, and bodies (as described above), fractions that were analyzed separately for δ^2^H. All samples were ground to a fine powder, and 350 μg of each was weighed into a 3.5 mm × 5 mm silver capsule and then analyzed by coupled pyrolysis gas isotope ratio mass spectrometry. Samples and standards were pyrolyzed at 1400°C in an alumina:glassy carbon reactor (Thermo-Finnigan TC/EA) producing H_2_ gas which was separated chromatographically in a 0.6-m molecular sieve gas chromatography (GC) column and analyzed for stable isotope composition using an isotope-ratio mass spectrometer (Thermo-Finnigan TC/EA and Delta^PLUS^-XL ™, Bremen, Germany). Non-exchangeable stable hydrogen isotopic ratios (^2^H/^1^H) of the Japanese beetles were reported in delta (δ) notation in parts per thousand (per mil, ‰) deviation from the VSMOW on the VSMOW-SLAP standard scale (Vienna Standard Mean Ocean Water-Standard Light Antarctic Precipitation) where δ^2^H = [(^2^H/^1^H_sample_) / (^2^H/^1^H_standard_) − 1]. In order to determine the δ^2^H of non-exchangeable H, we used the comparative equilibration method of [38]. Three keratin laboratory reference materials from Environment Canada CBS (Caribou hoof, -197.0 ‰), KHS (Kudu horn, -54.1 ‰), and Keratin SJ (-121.6 ‰), were used to normalize the results. CBS and KHS are described in [30]. Keratin SJ is Spectrum Keratin Powder Lot # SJ1400 from Spectrum Chemical Manufacturing Corporation, #K3030, Lot SJ1400 (Len Wassenaar, personal communication). The standard deviation for repeated analyses of an internal standard was consistently < 0.2 ‰.

### Potential Sources of Origin

For the beetles captured at PDX, we assessed potential source regions using information about the status of the Japanese beetle invasion, as reported in the U.S. Japanese Beetle Harmonization Plan [15], updated June 2013, and supplemented with information from state and university extension agencies about specific reports of Japanese beetle captures [12–14,16,17]. We focused on domestic sources only, because cargo air traffic to PDX is nearly exclusively domestic, as are the closest well-established populations of the Japanese beetle. Potential source regions included counties assigned to category 3 (Partially or Generally Infested) or to category 2 (Uninfested or Partially Infested) of the JB Harmonization Plan. Counties assigned to either category 1 (uninfested, under quarantine for import) or category 4 (not known to be infested) were not considered to be potential source regions. In Fig 1, areas assigned to categories 1 or 4 but with more recent reports of occasional captures of Japanese beetles are identified as sites experiencing potential invasion pressure (yellow regions), but these were not considered potential sources in the model. Major airports within each source region were identified and air traffic volume was recorded from publicly available data from the Federal Aviation Administration [39]. For all counties considered possible sources and for beetles whose isotope signatures identified the county as a possible source, the probability of a particular source county was weighted by air traffic from major airports. We also relaxed this prior, testing the model with equal probabilities of origin from all airports regardless of air traffic, an approach that assessed how well the stable isotope data could alone predict origin.

### Monitoring and Control Measures

The Oregon Department of Agriculture (ODA) implements a state-wide detection program for the Japanese beetle each year to prevent its establishment in the state. Japanese beetle traps are placed at high-risk areas throughout the state, including major transportation hubs. The greatest density of traps is placed at PDX. Traps are deployed regularly at air cargo sites and other sites around PDX and the surrounding area, and sites are sprayed each year with pesticides where adult beetles have been trapped. Records from these trapping efforts and pesticide applications were provided by the ODA for the work presented here. Aircraft inspections began in 1991. On average, 45 aircraft per year are inspected for Japanese beetles by ODA employees; the inspections report the number of beetles found and whether the beetles found were alive or dead. All data are included in the Supporting Information files: the isotope turnover experiment (S1 and S2 Datasets), the field-collected beetle specimens from voucher and PDX trapping collections (S3 Dataset), and data on trapping and spraying efforts (S4 Dataset).

### Bayesian Modeling

**Fig 2:**
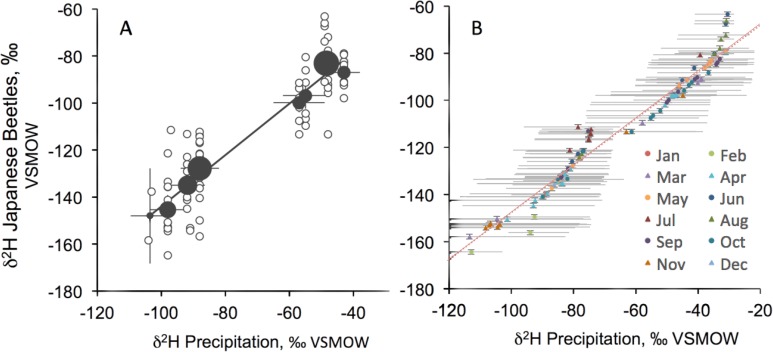
**A) Relationship between δ**^**2**^**H in Japanese beetles and the δ**^**2**^**H of mean annual precipitation from the region where beetles were collected.** Small open symbols represent individual beetle collections. Grey circles are means of beetle samples from sites of similar δ^2^H in precipitation, with the size of the grey circle scaled to the number of samples from the site. Vertical and horizontal bars indicate 95% confidence intervals. B) Relationship between δ^2^H in Japanese beetles and the posterior estimate of monthly δ^2^H value of precipitation, with the most important month of year shown in color codes. Horizontal bars are 95% credible intervals. For both A and B, whole beetles were used for the δ^2^H analysis.

We constructed a hierarchical Bayesian model to estimate the time elapsed between arrival and capture and potential sources of origin for each beetle trapped at PDX. The model includes 1) an isotope mixing model for beetles collected at or near PDX to estimate potential sources of origin compiled by county (Fig 1), selected based on the status of the Japanese beetle invasion within the conterminous U.S.A., and weighted by air traffic through major airports; 2) latent variable models for each beetle’s unknown source of origin and time-since-arrival; 3) a beetle end-member model that incorporates the observed relationship between δ^2^H of precipitation and δ^2^H of the voucher beetles (see Fig 2); and 4) an isotope mixing model that describes the change in beetle δ^2^H after experimental relocation to a novel isotope environment. The model explicitly accounts for multiple sources of variation and uncertainty associated with observations measured with error and predicted quantities produced with error.

#### Mixing model for PDX beetles

For each beetle *i* (*i* = 1, 2, …, 101) collected at or near PDX, we assume that the observed δ^2^H value of the beetle is normally distributed:

$$\delta^{2}{\text{H}}_{i}∼Normal\left(\mu \delta_{i},\sigma_{i}^{2}\right)$$ (1.1)

where μδ_*i*_ is the expected δ^2^H value and σ_*i*_^2^ is the residual error variance for beetle *i*. The expected value is based on a linear mixing model that assumes that the δ^2^H value of each beetle is a mixture of (1) a beetle’s δ^2^H value from its potential source region (origin), denoted by δ^2^H_Src_, and (2) the δ^2^H value of a beetle equilibrated with the PDX environment, denoted by δ^2^H_PDX_. Thus, μδ_*i*_ is given by:

$$\mu \delta_{i}=p_{i}\cdot \delta^{2}{\text{H}}_{\text{PDX},d(i)}+(1-p_{i})\cdot \delta^{2}{\text{H}}_{{\text{Src}}_{i},d(i)}$$ (1.2)

where *p* is the relative contribution (0 ≤ *p* ≤ 1) of the PDX “end-member,” *d*(*i*) denotes the date that beetle *i* was sampled, and Src_*i*_ denotes beetle *i*’s (unknown) source region. The date-specific end-members (δ^2^H_PDX,*d*(*i*)_ and $\delta^{2}{\text{H}}_{{\text{Src}}_{i},d(i)}$) were computed as a weighted average of the monthly values predicted for the past 90 days (a period selected to encompass the life-span of an adult Japanese beetle [5]):

$$\delta^{2}{\text{H}}_{\text{PDX},d(i)}=\sum_{j=0}^{3}w_{j,i}\delta^{2}{\text{H}}_{\text{PDX},m(i)-j}$$ (1.3)

where *w*_*j*,*i*_ is the weight associated with month index *j* relative to *m*(*i*), the month of collection, *m*, for beetle *i* (*j* = 0 = month of collection, *j* = 1 = previous month, etc.); the *w*_*j*,*i*_’s are computed as the proportional number of days (out of 90) that fall within each month period *j*. A similar equation is applied for $\delta^{2}{\text{H}}_{{\text{Src}}_{i},d(i)}$.

We also employed a mixing model for σ_*i*_^2^ to account for the possibility that the residual variance differs among sources:

$$\sigma_{i}^{2}=p_{i}\cdot \sigma_{\text{PDX}}^{2}+(1-p_{i})\cdot \left(\sigma_{\text{Source}}^{2}{\text{I}}_{{\text{Src}}_{i}<58}+\sigma_{\text{Composite}}^{2}{\text{I}}_{{\text{Src}}_{i}=58}\right)$$ (1.4)

where $\sigma_{\text{PDX}}^{2}$ is the variance in δ^2^H among beetles at or near PDX, $\sigma_{\text{Source}}^{2}$ is variance in δ^2^H among beetles originating from one of the 57 possible source regions, and $\sigma_{\text{Composite}}^{2}$ is variance in δ^2^H among beetles originating from a composite region whose δ^2^H values are not represented in the observed sources (see below). The indicator variable, I_X_, returns 0 if the logical function X is false, and 1 if X is true.

Based on the isotope turnover experiment, we modeled the relative contribution of the PDX end-member based on how long the beetle has been at or near PDX (i.e., time-since-arrive, or Time). In particular,

$$p_{i}=\max\left(0,\min\left(1,a+b\cdot {\text{Time}}_{i}\right)\right)$$ (1.5)

where *a* and *b* are parameters describing the rate of change in a beetle’s δ^2^H value following introduction to a new environment. T_0_ = -*a*/*b* + 14 is the number of days before the δ^2^H of the beetle starts to shift in response to a new environment (i.e., days for which *p* = 0), and T_1_ = (1-*a*)/*b* + 14 is the number of days it takes for the beetle to equilibrate with its new environment (i.e., when *p* = 1).

#### Latent beetle quantities

The above, beetle-specific isotope mixing model requires Time_*i*_ (time-since-arrival to PDX) and Src_*i*_ (potential source region) for each beetle, but these quantities are latent (i.e., unknown). We implemented a latent variable model for each. Based on information about Japanese beetle invasion and air traffic, we assumed that there were 57 potential source areas. The δ^2^H of the precipitation from these source areas could not account for the high δ^2^H values that were measured for ∼20% of the beetles collected at or near PDX. Thus, we introduced a 58^th^ composite region to account for an unknown source. For each beetle, we assume the simplest path from one potential source region to PDX. We specified an informative prior for Src_*i*_ such that for source region *j* (*j* = 1, 2, …, 58):

$${\text{Src}}_{i}=j\;\text{with probability}\;\pi_{j}$$ (1.6)

where π_*j*_ is proportional to the annual number of flights arriving at PDX from region *j*, and π_58_ is equal to 0.20 for the composite region. Eq 1.6 serves as an informative prior for the unknown Src_*i*_’s; the posterior solution distribution for each beetle’s Src may deviate from this prior once all data components and model assumptions are considered.

To constrain the estimates of Time_*i*_, we employed a hierarchical prior that beetles collected during the same year are likely to have similar days-since-arrival:

$${\text{Time}}_{i}∼LogNormal\left(\mu {\text{Time}}_{yr(i)},\sigma_{{\text{Time}}_{yr(i)}}^{2}\right)\text{I}(0.5,60)$$ (1.7)

where *yr*(*i*) denotes sampling year associated with beetle *i*, and I(0.5, 60) indicates that the lognormal distribution is truncated at 0.5 and 60 days to avoid unrealistically extreme values for Time_*i*_ (the “isotope turnover experiment” indicated that beetles equilibrate with their new environments well before 60 days). The arguments of the lognormal distribution (μTime and $\sigma_{\text{Time}}^{2}$) take on values on the log-scale and are assumed to vary by year.

#### δ^2^H end-members

The end-members represent the δ^2^H value of beetles equilibrated with the PDX environment (δ^2^H_PDX_) and with the source environment (δ^2^H_Src_). These are not known directly, but are informed by the δ^2^H values of precipitation in each location, and the relationship between beetle δ^2^H and precipitation δ^2^H based on the voucher specimens (see Fig 1). Following Eq 1.1, for each beetle voucher specimen *v* (*v* = 1, 2, …, 100), we assume:

$$\delta^{2}{\text{H}}_{v}∼Normal\left(\mu \delta_{{\text{VS}}_{v}},\sigma_{\text{VS}}^{2}\right)$$ (1.8)

where is the $\mu \delta_{{\text{VS}}_{v}}$ is the expected δ^2^H value for beetle *v*, and $\sigma_{\text{VS}}^{2}$ describes the residual error variance.

We modeled beetle δ^2^H based on the δ^2^H of the precipitation of each known collection location, such that the mean beetle δ^2^H is given by:

$$\mu \delta_{{\text{VS}}_{v}}=\Delta +\delta^{2}{\text{H}}_{{\text{Ppt}}_{l(v),m(v)}}$$ (1.9)

where Δ is the offset between beetle and precipitation δ^2^H, and δ^2^H_Ppt_ is the “true” δ^2^H value of precipitation, indexed by *l*(*v*), the location (*l*) associated with specimen *v*, and *m*(*v*), the month (*m*) during which specimen *v* was collected. *l*(*v*) is known, *m*(*v*) is not, so *m*(*v*) is treated as a latent variable, with a prior such that each of the 12 possible months (Jan, Feb, …, Dec) is equally probable.

The δ^2^H values describing the PDX and potential 58 source end-members (δ^2^H_PDX_ and δ^2^H_Src_, Eq 1.2) are computed based on the δ^2^H of precipitation in each area and the estimated offset derived from the voucher specimen model (Eq 1.9) such that the monthly end-member values are:

$$\begin{array}{c} \delta^{2}{\text{H}}_{{\text{PDX}}_{m}}=\Delta +\delta^{2}{\text{H}}_{{\text{Ppt}}_{\text{PDX},m}}\\ \delta^{2}{\text{H}}_{{\text{Src}}_{m}}=\Delta +\delta^{2}{\text{H}}_{{\text{Ppt}}_{\text{Src},m}} \end{array}.$$ (1.10)

The voucher data and precipitation data inform the offset (Δ); uncertainty in Δ is propagated to Eq 1.10 by modularizing the voucher-precipitation model [40–42].

We employed a Berkson-type model [43,44] to quantify uncertainty in the true δ^2^H of precipitation, expected to be normally distributed around location-specific estimates:

$$\delta^{2}{\text{H}}_{{\text{Ppt}}_{l,m}}∼Normal\left(\delta^{2}{\hat{H}}_{{\text{Ppt}}_{l,m}},{\hat{\sigma }}_{{\text{Ppt}}_{l}}^{2}\right)$$ (1.11)

where $\delta^{2}{\hat{H}}_{{\text{Ppt}}_{l,m}}$ are the predicted δ^2^H values, and ${\hat{\sigma }}_{{\text{Ppt}}_{l}}^{2}$ are the associated variances, both obtained from an on-line isotopes-in-precipitation calculator [26,37], which provides month-specific estimates ($\delta^{2}{\hat{H}}_{{\text{Ppt}}_{l,m}}$) and annual standard deviations for each location. We computed the variance describing variation in monthly δ^2^H (${\hat{\sigma }}_{{\text{Ppt}}_{l}}^{2}$) based on the formula for the variance of a mean ($\bar{x}$) of *N* random variables [45]:

$$\text{Var}(\bar{x})=\frac{1}{N^{2}}\left(\sum_{i=1}^{N}\text{Var}(x_{i})+2\sum_{i=1}^{N-1}\sum_{j>i}^{N}\text{Cov}(x_{i},x_{j})\right)=\frac{1}{N^{2}}\left(N\cdot \sigma^{2}+2\cdot \sigma^{2}\sum_{i=1}^{N-1}\sum_{j>i}^{N}\rho_{i,j}\right)$$ (1.12)

Eq 1.12 assumes that Var(*x*_*i*_) = σ^2^ (i.e., a common variance) for all *i* = 1, 2, …, *N*. Cov(*x*_*i*_,*x*_*j*_) is the covariance between *x*_*i*_ and *x*_*j*_, and *ρ*_*i*,*j*_ is the correlation coefficient. Here, *x*_*i*_ is the δ^2^H of precipitation in month *i* (for a given location), for *N* = 12 months, and σ^2^ is the variance in the monthly δ^2^H values (i.e., ${\hat{\sigma }}_{{\text{Ppt}}_{l}}^{2}$ in Eq 1.11). The variance associated with the annual values, $\text{Var}(\bar{x})$, is provided by the on-line isotopes in precipitation calculator (OPIC, [26,37]). The correlation, *ρ*_*i*,*j*_, was estimated by computing Pearson correlations between the monthly δ^2^H of precipitation for the 72 locations (57 source locations plus 15 voucher locations), which varied from *ρ* = 0.819 (Jan vs. Aug) to *ρ* = 0.999 (Apr vs. Jun), with an average *ρ* of 0.961 (based on 66 pairwise correlations). Plugging in values for *N* and each *ρ*_*i*,*j*_ into Eq 1.12, the monthly variance (${\hat{\sigma }}_{{\text{Ppt}}_{l}}^{2}$) can be estimated as a function of the location-specific (*l*) annual variance:

$${\hat{\sigma }}_{{\text{Ppt}}_{l}}^{2}=1.03745\cdot \text{Var}(\delta^{2}{\text{H}}_{{\text{Ppt}}_{l}})$$ (1.13)

Given the high *ρ* values, the monthly variances are slightly higher than the annual variances.

#### Mixing model for isotope turnover experiment

The isotope turnover experiment informs the parameters *a* and *b* in Eq 1.5 For beetle *j* (*j* = 1, 2, …, 62) in this experiment, we assume a likelihood similar to Eqs 1.1 and 1.11 such that:

$$\delta^{2}{\text{H}}_{j}∼Normal\left(\mu \delta^{2}{\text{H}}_{{\text{Exp}}_{j}},\sigma_{{\text{Exp}}_{g(j)}}^{2}\right)$$ (1.14)

where $\mu \delta^{2}{\text{H}}_{{\text{Exp}}_{j}}$ is the predicted δ^2^H value for beetle *j*, and $\sigma_{{\text{Exp}}_{g(j)}}^{2}$ is the residual variance for “group” *g* associated with beetle *j* (groups are defined below). Based on the experimental data, the predicted δ^2^H of beetle *j* ($\mu \delta^{2}{\text{H}}_{{\text{Exp}}_{j}}$) is expected to reflect the source environment for 14 days following relocation, with complete equilibration with the new environment after 35 days, and intermediate values between 14 and 35 days. Thus, the model for $\mu \delta^{2}{\text{H}}_{{\text{Exp}}_{j}}$ is:

$$\mu \delta^{2}{\text{H}}_{{\text{Exp}}_{j}}=\{\begin{array}{ccc} \delta^{2}{\text{H}}_{\text{Init}} & if & {\text{Time}}_{{\text{Exp}}_{j}}\leq 14\\ \delta^{2}{\text{H}}_{\text{Init}}+q_{j}(\delta^{2}{\text{H}}_{\text{Final}}-\delta^{2}{\text{H}}_{\text{Init}}) & if & 14\leq {\text{Time}}_{{\text{Exp}}_{j}}\leq 35\\ \delta^{2}{\text{H}}_{\text{Final}} & if & {\text{Time}}_{{\text{Exp}}_{j}}\geq 35 \end{array}$$ (1.15)

where δ^2^H_Init_ is the beetle δ^2^H value in the source environment, δ^2^H_Final_ is the value equilibrated with PDX, and *q*_*j*_ is the proportional contribution of the new isotope environment (δ^2^H_Final_) to beetle *j*’s δ^2^H value. The residual variance varies among the three categories of Time_Exp_ in Eq 1.15 (≤ 14 days, > 14 and < 35 days, or ≥ 35 days).

The proportional contribution of the new environment is expected to vary linearly with Time_Exp_, following Eq 1.5:

$$q_{j}=a+b\cdot {\text{Time}}_{{\text{Exp}}_{j}}$$ (1.16)

with *a* and *b* from Eq 1.5 (informed by the isotope turnover data), and the model described by Eqs 1.14 and 1.15 is modularized so that other datasets (such as the PDX beetle data) do not influence the *a* and *b* estimates. Uncertainty in these parameters (as quantified by their posterior distribution) is propagated to the model for *p*_*i*_ in Eq 1.5.

#### Implementation

We implemented the model in a Bayesian framework, specifying priors for all remaining unknown parameters (i.e., those not specified in Eqs 1.1–1.15, above). Standard deviation terms (σ) were assigned wide, uniform priors, *Uniform*(0,100), including the deviations for σ_PDX_, σ_Source_, and σ_Composite_ (Eq 1.4), σ_Time_ (Eq 1.7), σ_VS_ (Eq 1.8), and σ_Exp_ (Eq 1.14). We assigned a diffuse normal prior, *Normal*(0, 1×10^5^), to the offset between beetle δ^2^H and precipitation δ^2^H, Δ, in Eq 1.9. We assigned *Normal*(0, 1×10^7^) priors to the initial and final beetle δ^2^H values, δ^2^H_Init_ and δ^2^H_Final_, and to the *a* and *b* parameters in the isotope turnover experiment model in Eq 1.15; *b* was restricted to positive values. Finally, we assigned a *Normal*(0, 1×10^6^) prior to the year-specific mean time-since-arrival, μTime in Eq 1.7. Given the large variances of these normal priors, they are considered sufficiently vague.

We programmed the model in the Bayesian software package OpenBUGS [40]. Modularization of the voucher versus precipitation δ^2^H model (Eqs 1.8 and 1.9, the Berkson model for precipitation δ^2^H (Eqs 1.11–1.13), and the isotope turnover model (Eqs 1.14–1.16) was achieved by using the built-in cut function in OpenBUGS [40,42]. OpenBUGS employs Markov chain Monte Carlo (MCMC) to sample from the joint posterior of all unknown quantities. We ran three parallel MCMC chains with relatively dispersed starting values and at least 20,000 iterations. Convergence was evaluated with the built-in Brooks-Rubin-Gelman convergence diagnostic tool (chains converged by 5,000 iterations). The marginal posterior distribution for each quantity was summarized by its posterior mean and central 95% credible interval (CI), defined by the 2.5^th^ and 97.5^th^ percentiles. Annotated OpenBUGS code is provided as Supporting Information (S1 Text), as well as the data input file (S5 Dataset), and model results (S6 Dataset).

## Results

Hydrogen isotope signatures (δ^2^H) in Japanese beetle specimens collected from sites across the continental U.S.A. varied by region, and were correlated with δ^2^H of annual precipitation (Fig 2A, R^2^ = 0.81, S3 Dataset). The relationship improved when monthly data were used (R^2^ = 0.97), and the latent month was estimated, which indicated that the months spanning April to October were most likely to have contributed to beetle δ^2^H (Fig 2B, S6 Dataset). Based on this relationship, the Japanese beetles are expected to be 47.9‰ lower in δ^2^H compared to precipitation (posterior mean and 95% CI for Δ = -47.9 [-51.3, -44.3]).

**Fig 3:**
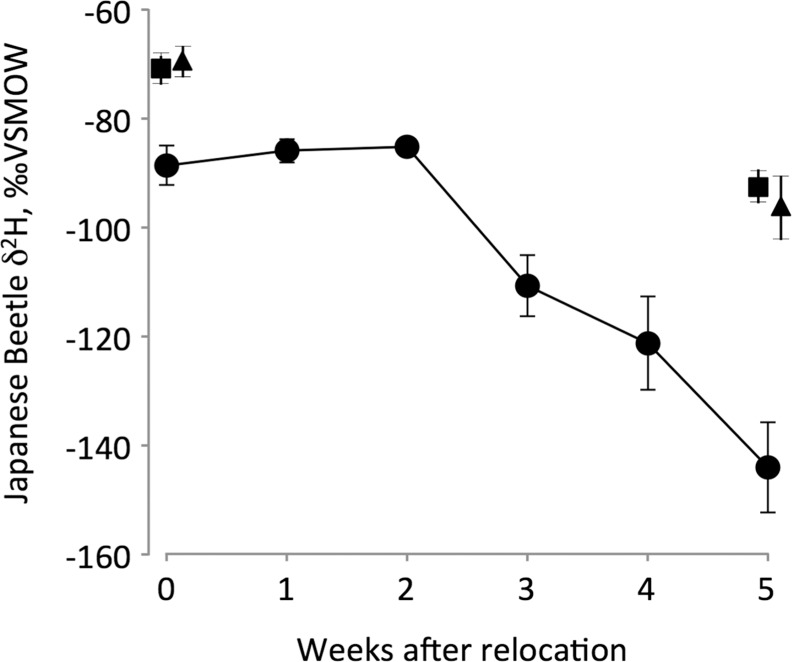
δ^2^H of Japanese beetles during a 5-week experiment in which beetles from Virginia were reared in terracosms with plant material and environmental water from Oregon. Symbols show means and bars two standard errors. Whole-body tissue samples were analyzed each week (circles), whereas wings (squares) and elytra (triangles) were sampled in week 0 and week 5.

The δ^2^H of beetles from Virginia experimentally reared in a terracosm with Oregon plants and water sources (isotope turnover experiment) began changing after about 14 days (T_0_ = 13.4 [7.5, 17.4], Fig 3, S1 Dataset). The δ^2^H values declined after that point at a rate of 4.7% per day (posterior estimate of slope, b, mean = 0.047, 95% CI = [0.031 to 0.063]). After about 35 days, beetles reached δ^2^H values expected for equilibrium with their new isotope environment (T_1_ = 35.6 [31.4, 42.2]). After five weeks, wings and elytra exhibited a smaller shift in δ^2^H compared to the rest of the body (Fig 3), likely indicating slower turnover of H through keratin and chitin. Still, the isotopic shift was apparent in these tissues, indicating that hydrogen incorporation into or synthesis of new keratin and chitin occurred during the 5-week experiment.

**Fig 4:**
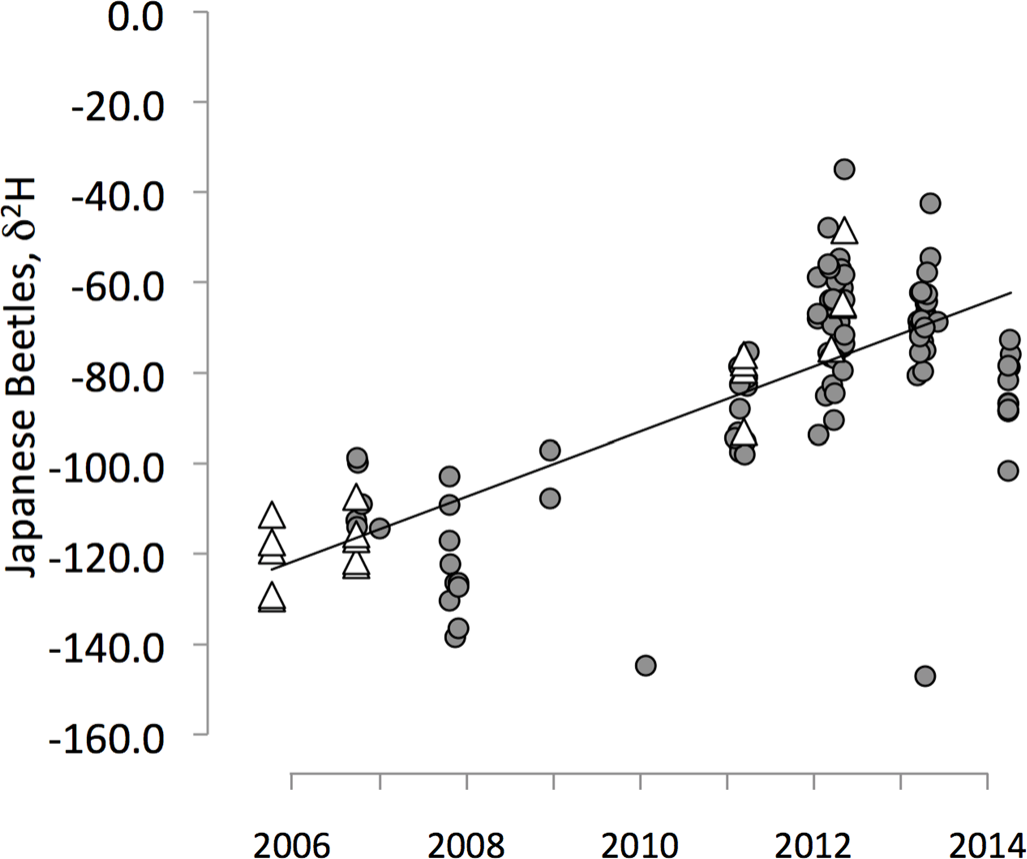
δ^2^H of Japanese beetles collected at the Portland International Airport (Oregon, USA) (circles) and in locations nearby (triangles) from 2006–2013. Increased trap densities around the airport have increased the probability of catching beetles as they fly from planes.

**Fig 5:**
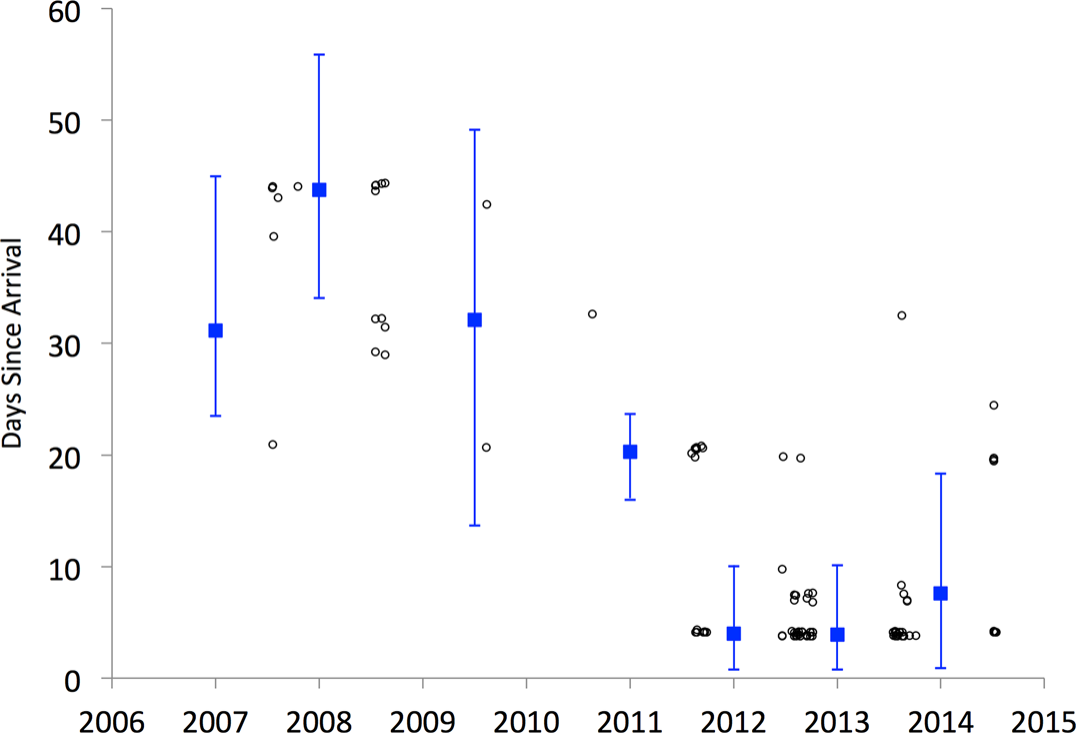
Estimated days between arrival and capture of each individual beetle captured at the Portland International Airport (open circles) and the average for each year (blue squares, with bars showing 95% confidence intervals; note year with combined data). The solid line shows the exponential fit for the temporal relationship.

The δ^2^H signatures of Japanese beetles collected in the area around the Portland International Airport (PDX) increased over time from 2007 to 2014 (R^2^ = 0.63, slope = 9.2‰ per year), initially reflecting the δ^2^H of organisms equilibrated with Portland precipitation, and later matching the δ^2^H of organisms from the eastern U.S.A. (Fig 4, S3 Dataset). These changes in the δ^2^H value of beetles trapped at PDX indicated a reduction in the average time-since-arrival over the sampling interval (Fig 5, S6 Dataset). For example, the average time-since-arrival varied from ∼ 44 days in 2008 to ∼ 4 to 8 days in 2012–2014 (Fig 5). Given that the beetles retain their source origin isotope signature for ∼14 days after arriving at PDX, it is difficult to resolve finer scale changes in time-since-arrival. Nevertheless, beetles collected in 2012–2014 were recent arrivals, whereas beetles collected early in the study (2007–2010) had been present longer before trapping, long enough that they may have begun to establish resident populations (95% CI for mean time-since-arrive contains 35 days). 2011 marks a transition period where beetles collected at PDX had arrived ∼20 days before capture (95% CI for mean time-since-arrival = [16, 24] days) (Fig 5).

**Fig 6:**
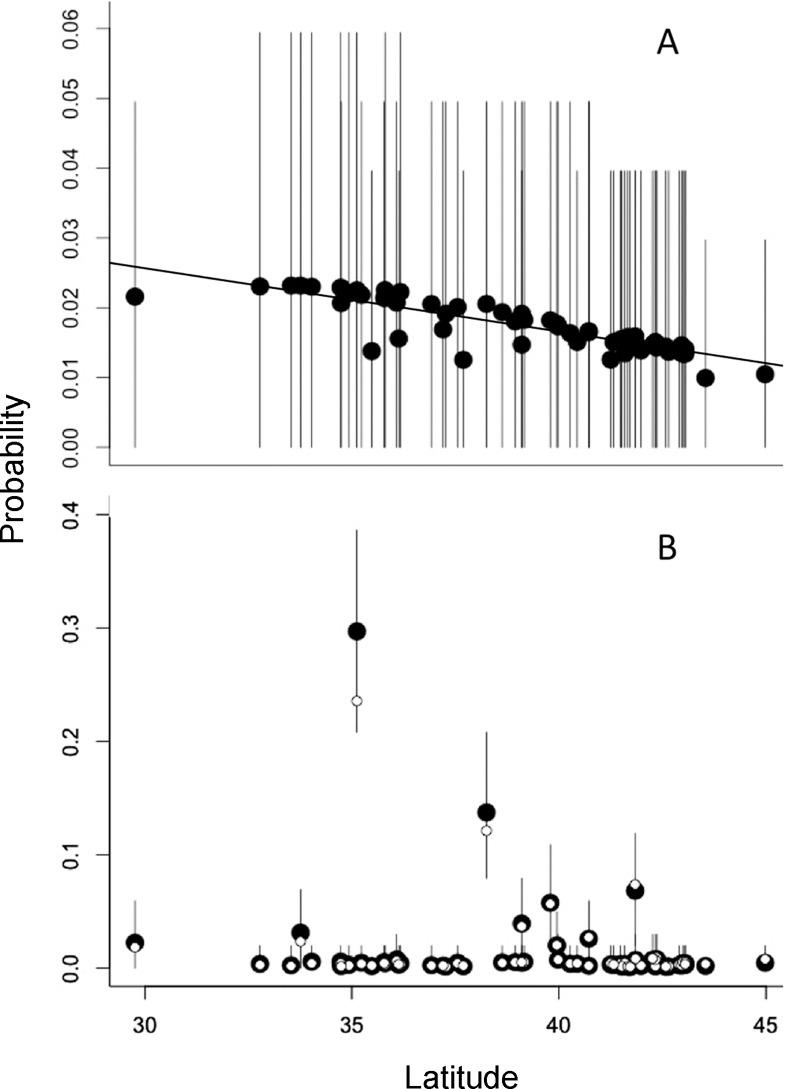
A. **Probability of specific locations as sources for Japanese beetles** arriving at PDX as a function of latitude, using the “flat prior” model where all cities are assigned an equal prior likelihood. Filled circles show the mean posterior estimate from the hierarchical Bayesian model and vertical lines show 95% credible intervals. The linear fit is the weighted regression (P<0.001, slope = -0.00091 ± 0.00007), where each data point is weighted by the inverse of the credible interval (thus, smaller credible intervals have more weight). B. Same as A, but using the hierarchical Bayesian model in which the probability of each city is proportional to the cargo air traffic density from [39]. The prior assignments are shown in small white circles. The weighted regression is not significant (P = 0.486, slope = -0.00044 ± 0.00064).

Assigning likelihoods to specific source regions of newly arrived Japanese beetles at PDX was possible with our hierarchical Bayesian model. The model run with the “flat prior”, which assigned equal likelihoods to each potential source city, resulted in a strong pattern: more southerly locations had higher probabilities of being sources (Fig 6A, weighted regression, P<0.001, S6 Dataset), though the credible interval around the probabilities for each potential source were large. In the model run including the airline traffic data, the probability of specific locations was more strongly predicted by the prior (i.e., air traffic density), than by the isotope data (Fig 6B). Including the isotope data tended to increase the value of the posterior compared to the prior for more southerly locations, whereas the posterior tended to decline compared to the prior for more northerly latitudes (Fig 6B): the difference (posterior–prior) declined with latitude (slope = -0.00072 ± 0.00032, P = 0.031). Together, the δ^2^H data indicate that the southeastern U.S.A. is the more likely source of Japanese beetles arriving at PDX.

**Fig 7:**
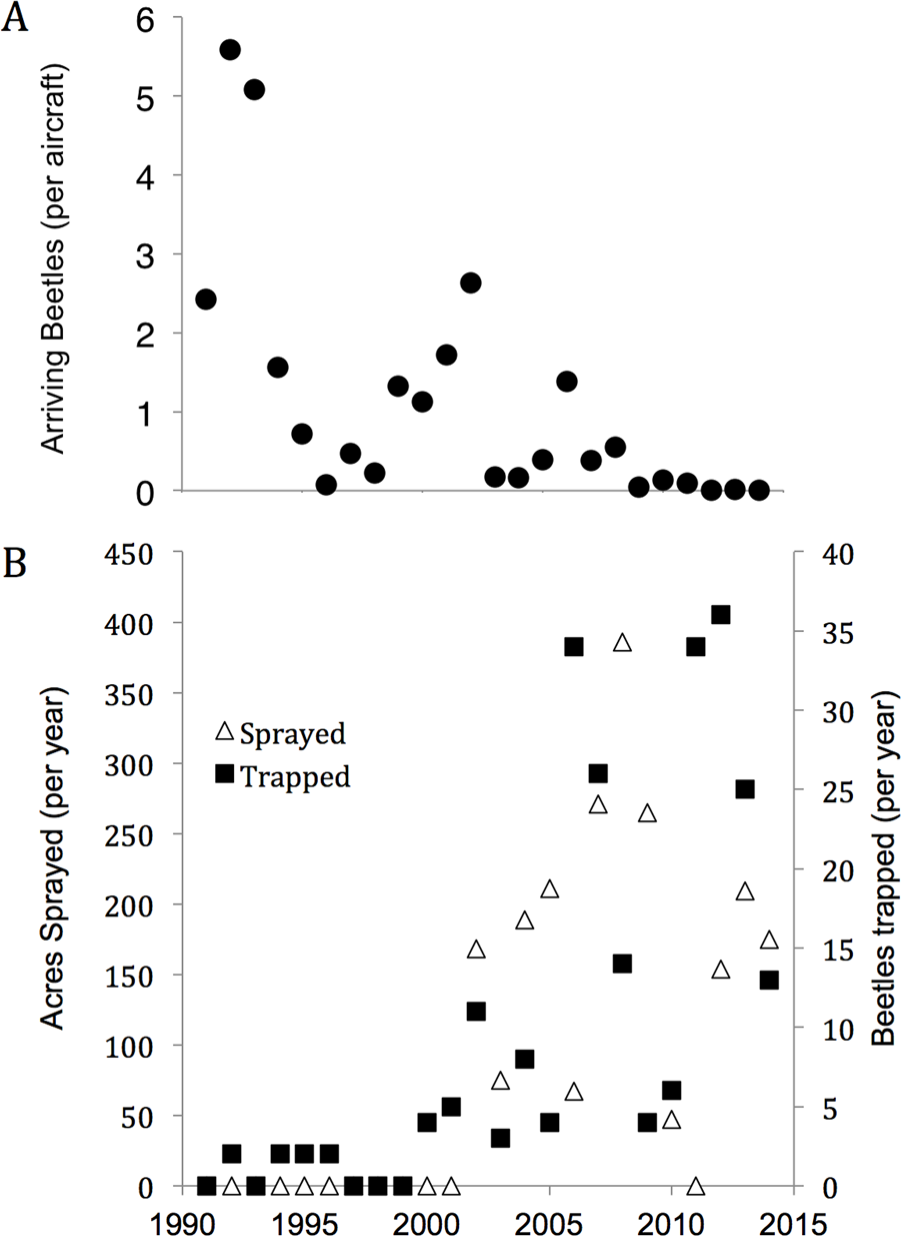
A. **Number of Japanese beetles encountered per aircraft inspected over time.** Inspections began in 1991 and an average of 45.1 aircraft were inspected per year. B. Number of Japanese beetles trapped per year, and number of acres sprayed per year with pesticides by the Oregon Department of Agriculture in the vicinity of the Portland International Airport (PDX).

The number of beetles per cargo aircraft at PDX declined exponentially from 1991 to 2014 at a rate of 15% per year (Fig 7A, S4 Dataset). From 2003 to 2014, air cargo traffic to PDX declined from approximately 90 to 50 cargo flight operations per day [46] (S4 Dataset). Efforts to control the Japanese beetle around PDX through pesticide application was higher during the sampling period (2006–2014) compared to the previous 15 years, and density of traps increased during this period as well, from 100 in the 1990s, to 1000 or more in the 2000s (S4 Dataset). The area sprayed each year did not exhibit any temporal trend from 2006 to 2014 (Fig 7B). Similarly, the numbers of beetles trapped per year did not exhibit strong temporal trends over the sampling period (2006–2014) but was higher during this period compared to the previous 15 years (1991–2005, Fig 7B), coincident with the increase in trap deployment.

## Discussion

Japanese beetles collected from established populations in the eastern U.S.A. were consistently higher in δ^2^H compared to individuals collected from the western U.S.A. (Fig 2), indicating fidelity between δ^2^H of body tissues and δ^2^H of precipitation at the site of origin for the Japanese beetle, consistent with numerous other studies that have documented such relationships between δ^2^H of precipitation and insect tissues [21,24,28]. The relationship we found for Japanese beetles has a similar coefficient of determination and slightly larger offset (-47.9‰, R^2^ = 0.81) between beetle δ^2^H and precipitation δ^2^H compared to past analyses for butterfly wings in Europe (-40.6‰, R^2^ = 0.87 [28]) and dragonfly wings in North America -42.5‰, R^2^ = 0.75 [31]), though overall the relationships are quite similar. Our results confirm that, in regions with sufficient geographic variation in the δ^2^H value of precipitation, stable isotopes are useful for tracing the natal origins of organisms, including butterflies [19], birds [32,47], bats [48], and invasive beetles (Fig 2). Our work also confirms the potential for stable isotopes to provide information about the more recent movements of organisms [28,49], which could prove valuable in tracking and managing invasive organisms [50].

For invasive species, isotopes can provide information complementary to other techniques. Molecular signals can resolve geographic patterns of invasive species’ range expansions as invasions proceed, distinguishing invaders from different source populations [51], as long as the populations have been separate for enough generations to develop genetic differences. For more rapidly expanding invasive species, genetic differentiation among populations may be absent and therefore not useful in identifying proximate sources [52], and, molecular signals provide little information about the short-term temporal dynamics of migrating organisms relevant to the time-scale of biological invasions. In contrast, the stable isotope composition of organisms can change within the lifetime of an individual, depending on the timescale of element turnover through body tissues [53] or body water [54]. Compared to molecular genetic tools, stable isotopes can reveal information about the recent origin of invasive organisms on a much finer timescale, likely more suitable to the timescale of decisions about controlling populations of invasive organisms. For example, δ^2^H analyses showed that populations of the marmalade hoverfly (*Episyrphus balteatus*) occurring in agricultural fields in France comprise both locally established individuals and new arrivals from more southern regions [55]. This species of hoverfly eats aphids so is important in biological control of an agricultural pest. The finding of low immigration indicates that focusing management on overwintering populations may be the most effective means to ensure continued biocontrol from this species [55]. For pests, as in our study, using stable isotopes can also help identify the immediate origins of individual organisms, whether they are locally established or intercepted vagrants.

The hierarchical Bayesian model did not clearly distinguish specific source regions of Japanese beetles, likely because of the many disparate sources of Japanese beetles throughout the eastern U.S.A. The resolution of the model could be improved in several ways: (1) finer resolution between the development of the beetles and the tissue δ^2^H, possibly resulting in a better regression based on monthly precipitation; (2) a model that more fully accounts for the beetles’ ontogeny, tracking, for example, the higher likelihood that earlier arrivals at PDX come from more southern sites with warmer climates and thus earlier beetle emergence; (3) including beetle tissues of varying turnover time and using the differences among tissues in δ^2^H to provide even greater temporal resolution; and (4) including other tracers that exhibit more geographic variation among potential source regions (possibly genetic markers, or other elements and isotopes). Despite the weak spatial resolution, the estimates of time since arrival were robust (R^2^ = 0.92 and slope = 0.92, for a comparison of time since arrival predicted between model runs with and without the air traffic prior). There is less geographic variation in δ^2^H among potential source regions compared to the difference in δ^2^H between all eastern source populations and sites in the western U.S.A. vulnerable to invasion. For this reason, estimates of arrival time were more precise that were estimates of geographical origin.

The increasing δ^2^H signatures of Japanese beetles captured around PDX (Fig 4) indicate that individuals collected during the earlier years (2006–2009) of control efforts may have been part of an establishing population, partially or fully equilibrated with the new (Portland) isotopic environment. By contrast, organisms captured more recently (2010–2014) were newer arrivals (Fig 5), still reflecting the isotopic signature of the source populations from the likely origin sites in the eastern USA (Fig 2). The shift in δ^2^H signatures toward source populations coincident with multiple years of pesticide ground treatments suggests that these management strategies in the area surrounding PDX has helped prevent the establishment of a local viable population. The decline in the frequency of beetles found on inspected aircraft (Fig 7) is consistent with more effective control measures at the source populations [56], especially given the decline in cargo air traffic to PDX from 2003 to 2014 [46]. Together, these indicate a reduction in the invasion pressure from cargo aircraft to PDX over this time period. Despite better control at source populations, new individuals continue to arrive with distinctly eastern δ^2^H signatures. Increased trap deployment may have reduced the time between the beetles’ arrival and trapping. δ^2^H appears to have been an independent and sensitive indicator of the status of the invasion. While it is difficult to attribute the change in δ^2^H over time to specific causes, the decline is consistent with control measures that have reduced the time between the arrival and capture of potentially invading individuals.

The anomalously low δ^2^H signatures for some individual Japanese beetles captured in 2010 and 2013 were statistical outliers for the populations of beetle captures during those years, though they fell within the range of δ^2^H values expected for organisms equilibrated with the PDX environment. In the world of invasions, outliers may be the few individuals required to establish a viable population [57]: these low δ^2^H values indicate that these individuals were present in the region long enough to equilibrate with the new environment. Given the heterogeneous distribution of Japanese beetle populations across the landscape, a low number of trapped beetles could indicate the presence of a viable population [58].

Our finding that δ^2^H of beetle tissues shifted after experimentally controlled migration from Virginia to Oregon (Fig 3) demonstrates that bulk tissue hydrogen isotope analysis can resolve recent movements of this organism: the shift in δ^2^H began after two weeks in the novel environment, was statistically significant after three weeks, and changed over a period of five weeks before reaching the expected δ^2^H value of the new environment. Individual Japanese beetles that preserve a clear δ^2^H signature of a distant source population are likely to be new arrivals, where ‘new’ for this system means having arrived within the preceding two weeks. Compared to the shift in beetle body δ^2^H after five weeks, the smaller shift in δ^2^H in wings and elytra by week five is consistent with these tissues being composed of keratin and chitin, thought to be stable after synthesis; yet, the decline in δ^2^H suggests that hydrogen exchange or new tissue formation occurred in these organisms during the experiment (Fig 3B). Combining δ^2^H analysis of stable tissues to record natal origin and of tissues that turn over more rapidly to record the timing of relocation would likely provide more power in resolving both the origins and movements of invasive organisms.

Isotope monitoring can guide management strategies: any shift toward local precipitation would be evidence that colonists are beginning to establish a new population, and would justify more intensive local control measures. By contrast, persistent trapping of individuals with the δ^2^H value consistent with the source populations would support increased inspection and prevention of colonists’ being able to board the aircraft in the first place. Continued trapping and monitoring the isotopic signatures of these potentially invasive organisms can provide managers with critical information about the status of the invasion. In general, our results point to the utility of isotope sentinels for invasive species: in cases where the δ^2^H of precipitation differs between source populations and sites at risk for invasion, monitoring captured individuals for isotopic composition can distinguish between recent arrivals and establishing populations.

## Supporting Information

S1 Dataset Data from the isotope turnover experiment in which beetles were experimentally transplanted to Oregon from their native environment in Virginia and grown in a terrarium. Beetle samples were collected weekly for δ^2^H determinations to determine the shift in δ^2^H over time. Excel workbook with 2 worksheets.
(XLSX)

S2 Dataset Mass of Japanese beetle wings, elytra, and remainder of the body. These masses were used to reconstruct whole-beetle δ^2^H values in the isotope turnover for weeks 1 and 5, cases where each beetle part was analyzed separately for δ^2^H. The δ^2^H value of the whole beetle sample was then calculated by mass balance. Excel workbook with 2 worksheets.
(XLSX)

S3 Dataset Japanese beetle δ^2^H values from samples collected around the conterminous U.S.A. and from within the study region, the area around the Portland International Airport (PDX, Portland, OR, U.S.A.). Excel workbook with 2 worksheets.
(XLSX)

S4 Dataset Control measures taken by the Oregon Department of Agriculture to limit the invasion of the Japanese beetle, focusing on the Portland International Airport (PDX, Portland, OR, U.S.A.) as a potential point of entry. Data include the numbers of beetles found during aircraft inspections, the numbers of beetles trapped in the field in the PDX region, the ground area treated with pesticide applications, the number of traps deployed each year in the PDX region, and air cargo traffic data available from the Port of Portland website. Excel workbook with 3 worksheets.
(XLSX)

S5 Dataset Input data required to run the hierarchical Bayesian model. See readme file for more information. Excel workbook with 7 worksheets.
(XLSX)

S6 Dataset Output data from the hierarchical Bayesian model. See readme file for more information. Excel workbook with 9 worksheets.
(XLSX)

S1 Text The hierarchical Bayesian model code, written in the program OpenBUGS. PDF file with 6 pages.
(PDF)
