# Supplementary material for: Hydrogen Isotopes as a Sentinel of Biological Invasion by the Japanese Beetle, Popillia japonica (Newman)
Source: PLoS One. 2016 Mar 9;11(3):e0149599. doi: 10.1371/journal.pone.0149599 (PMC4784742; doi:10.1371/journal.pone.0149599)
Supplement: S1 Text — PDF file with 6 pages. (PDF) [file pone.0149599.s007.pdf]

Hierarchical Bayesian Model

Hydrogen Isotopes as a Sentinel of Biological Invasion by the Japanese Beetle, *Popillia japonica* (Newman)

Model Code Written by Professor Kiona Ogle

Informatics and Computing Program, Center for Ecosystem Science and Society and Department of Biological Sciences, Northern Arizona University, Flagstaff AZ 86011, USA

23 November 2015

Model Program: OpenBUGS, described in Lunn D, Spiegelhalter D, Thomas A, Best N (2009) The BUGS project: Evolution, critique and future directions. Statistics in Medicine 28: 3049-3067.

```
*Data components are in bold font
#Annotations

model{
    # Mixing model for individual beetles sampled in Portland:
    for(i in 1:Nbeetles){
        # Likelihood of d2H beetle data:
        dbeetle[i] ~ dnorm(mu[i],tau[i])
        # Generate replicated data for evaluating model fit:
        dbeetle.rep[i] ~ dnorm(mu[i],tau[i])

        # Compute squared error for evaluating model fit:
        sqdiff[i] <- pow(dbeetle[i]-dbeetle.rep[i],2)

        # Compute precision, where sig = std deviation.
        tau[i] <- pow(sig[i],-2)

        # Assume observation std dev is a mixture of a standard deviation associated
        # purely Portland beetles (p = 1) and beetles purely from other cities (p = 1)
        sig[i] <- p[i]*sigCity[1] + (1-p[i])*sigCityTemp[i]

        # Simple linear mixing model:
        # Beetle is a mixture of d2H value from PDX and from potential source city:
        mu[i] <- p[i]*dPDX[i] + (1-p[i])*dSourceCity[i]

        # Get the month (1,2,.., 12) associated with the date of collection (doc)
        mo[i] <- month[doc[i]]

        # Get month prior to date of collection; if month of collection = Jan, then
        # set previous (prior) month = December (month 12)
        prev.mo[i] <- (1-equals(mo[i],1))*(mo[i]-1) + equals(mo[i],1)*12
        # Get month that is two months prior to date of collection:
        prev2.mo[i] <- (1-equals(prev.mo[i],1))*(prev.mo[i]-1) +equals(prev.mo[i],1)*12

        # Weights associated with contribution of current month's precip (w[1,]),
        # previous month's precip (w[2,]), and 2-months ago precip (w[3,]), assuming an
        # average lifespan of 90 days:
        w[1,i] <- (doc[i]-(first.day[mo[i]]-1))/90
        w[2,i] <- (last.day[prev.mo[i]] - (first.day[prev.mo[i]]-1))/90
        w[3,i] <- 1-w[1,i]-w[2,i]

        # Compute d2H value of PDX beetles and beetles from source city by taking a
        # weighted average of current, past and 2-months ago values predicted from
        # precip relationshop; dsource[72] = d2H value for portland area (city
        # ID # 72). Include a "composite" city/source (Ncity + 1 = 58) that represents
        # the highest (heaviest) beetle d2H value (i.e., a beetle d2H value of -35
        # per mil).
```

```
    dPDX[i] <- w[1,i]*dsource[72,mo[i]] + w[2,i]*dsource[72,prev.mo[i]] +
                w[3,i]*dsource[72,prev2.mo[i]]

    dTempCity[i] <- w[1,i]*dsource[City[i],mo[i]] +
                w[2,i]*dsource[City[i],prev.mo[i]] +
                w[3,i]*dsource[City[i],prev2.mo[i]]

    # If the source city was 1, 2, ..., 57, then grab appropriate dTempCity value;
    # otherwise, use "composite" city value:
    dSourceCity[i] <- (1-equals(City[i],Ncity+1))*dTempCity[i] +
                      equals(City[i],Ncity+1)*(-35)

    # Likewise, if the source city is one of 1,2,..., 57, grab the standard dev
    # associated with "other source cities," otherwise, get the "composite"
    # city std dev (sigCity[3]).
    sigCityTemp[i] <- (1-equals(City[i],Ncity+1))*sigCity[2]+
                      equals(City[i],Ncity+1)*sigCity[3]

    # q is the contribution of the source city:
    q[i] <- 1-p[i]

    # p is the contribution of portland end-member, which varies with
    # time since beetle arrived at PDX (Time); intercept (a) and slope
    # (b) determined from a separate "calibration" analysis with the
    # experimental, isotope turn-over data:
    p[i] <- max(0,min(1, a + b*(Time[i]-14)))

    # Latent, unknow City origin (source) of beetle:
    # If City = 58 (=Ncity + 1), then this is the "composite" city
    City[i] ~ dcat(pcity.new[1:(Ncity+1)])

    # Latent, unknown time since arrival of beetle:
    # Treat Time as continuous, but restrict to 0.5 to 60 days;
    # Assume a hierarchical prior that allows for beetles within a given
    # year to "borrow strength" from each other, such that the mean and variance
    # associated with time-since-arrival varies by year:
    Time[i] ~ dlnorm(mu.Time[YrID[i]],tau.Time[YrID[i]])I(0.5,60)

    # Monitor the invasion status of each beetle
    # (0 = "false" or 1 = "true" for each)
    # Threshold is computed later.
    new.arrival[i] <-  step(Tthreshold[1]-Time[i])
    invading[i] <- step(Tthreshold[2]-Time[i])*step(Time[i]-Tthreshold[1])
    established[i] <- step(Time[i]-Tthreshold[2])

    # Keep track of which cities the beetles are coming from (1 = beetle came
    # from city):
    for(c in 1:Ncities){
       Source.City[c,i] <- equals(c,City[i])
       }
    }

# Total squared error, for model evaluation:
Dsum <- sum(sqdiff[])

# Vague priors for the standard deviation terms (1 = Portland,
# 2 = other source cities, 3 = composite city):
for(c in 1:3){
    sigCity[c] ~ dunif(0,100)
}

# Prior for probability of each source city using airline travel density data
for(i in 1:Ncity){
```

```
    # Original relative airline density for cities 1,2,.., 57:
    pcity.temp[i] <- pcity[i]
    # Rescaled relative airline density for cities 1,2,.., 57 (to account for
    # the composite city):
    pcity.new[i] <- pcity.temp[i]/sum.pcity
    }

# Prior for composite city; based on observation that about 1/4 of the PDX
# beetle d2H values are greater (less negative) than would be predicted
# from the maximum d2H precip values, across all cities.
pcity.temp[Ncity+1] <- 0.25

# Rescaled relative airline density for composite city:
pcity.new[Ncity+1] <- pcity.temp[Ncity+1]/sum.pcity
sum.pcity <- sum(pcity.temp[])

    # Population invasion status:
    # Overall proportion of beetles that are recent arrivals, invading,
    # established, and not established (i.e., new arrivals or invading):
    mean.arrival <- mean(new.arrival[])
    mean.invading <- mean(invading[])
    mean.established <- mean(established[])
    mean.notEstablished <- 1 - mean.established

# For all cities, account for uncertainty in precip d2H monthly values:
for(j in 1:Ncities){
    for(m in 1:12){
        # Berkson model for "errors in variables" associated with
        # city d2H values.
        # Generate potential city d2H values of precip given monthly estimates
        # and estimates of uncertainty:
        dppt.temp[j, m] ~ dnorm(dppt[j,m], tau.ppt[j,m])
        tau.ppt[j,m] <- pow(sd.monthly[j,m],-2)

        # Compute monthly standard deviation given annual stand dev (sd.ppt):
        sd.monthly[j,m] <- 1.01855*sd.ppt[j]

        # Cut the generated precip d2H value so other model components don't
        # feed back to adjust these values:
        dppt.true[j,m] <- cut(dppt.temp[j,m])
        }
    }

# Herbarium/voucher model to get estimate offset between
# precip d2H values and beetle d2H values.
for(j in 1:Nherb){
    # Likelihood of beetle voucher/herbarium d2H values:
    dherb[j] ~ dnorm(mu.herb[j], tau.herb)
    # Generate replicated data:
    dherb.rep[j] ~ dnorm(mu.herb[j], tau.herb)
    # Unknown date of collection (month):
    doc.temp[j] ~ dcat(p.doc.herb[1:12])
    doc.herb[j] <- doc.temp[j]
    # Regress the beetle d2H value (mu.herb) on the "true" precip
    # d2H value for the corresponding date-of-collection (month):
    mu.herb[j] <- a0 + b0*dppt.true[CityID[j], doc.herb[j]]
    herb.precip[j] <- dppt.true[CityID[j], doc.herb[j]]
    # Keep track of the month that each beetle is coupled to:
    for(m in 1:12){
        mo.herb[m,j] <- equals(doc.herb[j],m)
        }
    }
```

```
# Compute proportion of beetles "coupled" to each month:
for(m in 1:12){
   pherb.temp[m] <- mean(mo.herb[m,])
   }

# Prior probability of each month:
for(d in 1:12){
   p.doc.herb[d] <- 1/12
   }

# Relatively non-informative (flat) prior for stand dev associated with
# voucher d2H data:
tau.herb <- pow(sig.herb,-2)
sig.herb ~ dunif(0,100)

# Prior for the intercept ("offset") for the voucher d2H vs precip d2H regression:
a0 ~ dnorm(0,0.00001)
# Set the slope = 1, so intercept is interpreted as a pure offset:
b0 <- 1
# Use cut function such that uncertainty in coefficients is propagated
# to the PDX mixing model, but the other data sources do not change/update
# these parameters (note: cut is not needed for b0 since it is fixed):
a0.cut <- cut(a0)
b0.cut <- cut(b0)

# For each city, generate potential monthly d2H values of a beetle coming from
# city by propagating uncertainty from the voucher/herbarium vs precip d2H model:
for(j in 1:Ncities){
   for(m in 1:12){
      dsource[j,m] <- a0.cut + b0.cut*dppt.true[j,m]
      }
   }

# Lower time "threshold" at which p = 0:
Tthreshold[1] <- -a/b + 14
# Upper time "threshold" at which p = 1:
Tthreshold[2] <- (1-a)/b + 14

# Experimental model for isotope turn-over data:
# Fit regression model to experimental data for the mixing model
# that describes the effect of "time-since-arrival" on the isotope
# mixing proportion:

# Initial end-member (based on first 31 observations, time <= 14 days):
for(i in 1:31){
   dmix[i] ~ dnorm(dinit, tau.init)
   }
# Final end-member (for time = 35 days):
for(i in 53:62){
   dmix[i] ~ dnorm(dfinal, tau.final)
   }

# Priors for end-members (and standard deviations), and cut for
# propogating uncertainty:
dinit ~ dnorm(0,0.0000001)
dfinal ~ dnorm(0,0.0000001)
tau.init <- pow(sig.init,-2)
sig.init ~ dunif(0, 100)
tau.final <- cut(tau.init)
cut.dinit <- cut(dinit)
cut.dfinal <- cut(dfinal)

# For mixture data (i.e., for which time between 14 & 35 days):
```

```
for(i in 21:62){
    # compute pmix = proportion of final d2H value:
    pmix[i] <- (dmix[i] - cut.dinit)/(cut.dfinal - cut.dinit)
    # Implement "zeros-trick" to implement likelihood below:
    # pmix[i] ~ dnorm(mu.mix[i], tau.mix)
    zeros[i] <- 0
    LL[i] <- 0.5*log(tau.mix/(2*3.141593)) - (tau.mix/2)*pow(pmix[i] - mu.mix[i],2)
    phi[i] <- -LL[i] + C
    zeros[i] ~ dpois(phi[i])
    mu.mix[i] <- aa + bb*(Time.mix[i]-14)

    # Generate replicated data:
    pmix.rep[i] ~ dnorm(mu.mix[i], tau.mix)
    dmix.rep[i] <- pmix.rep[i]*(cut.dfinal - cut.dinit) + cut.dinit
    }
C <- 5

# Priors for linear portion of the d2H vs time regression;
# rescrict slope to be positive:
aa ~ dnorm(0,0.0000001)
bb ~ dnorm(0,0.0000001)I(0,)
# Use the cut function to propagate uncertainty:
a <- cut(aa)
b <- cut(bb)
tau.mix <- pow(sig.mix,-2)
sig.mix ~ dunif(0,100)

# Relatively non-informative, independent priors for parameters
# associated with the hierarchical model for the time-since-
# arrival model (for each PDX beetle):
for(y in 1:Nyr){
    mu.Time[y] ~ dnorm(0,0.000001)
    # Expected number of days-since-arrival (on regular scale of days):
    mu.Time.Days[y] <- exp(mu.Time[y] + 0.5*pow(sig.Time[y],2))
    sig.Time[y] ~ dunif(0,100)
    tau.Time[y] <- pow(sig.Time[y],-2)
    # Stand dev for number of days-since-arrival (on regular scale of days):
    sig.Time.Days[y] <- mu.Time.Days[y]*sqrt(exp(pow(sig.Time[y],2) - 1))
    }

# Compute the overall average contribution of Portland (pave)
# versus the "source city" (qave):
pave <- mean(p[])
qave <- mean(q[])

# Compute year-level population estimates:
# y = 1 [2007], 2 [2008], 3 [2009-2010], 4 [2011], 5 [2012], 6 [2013], 7 = [2014]
for(y in 1:Nyrs){
    # contribution of portland:
    p.year[y] <- mean(p[start[y]:end[y]])
    # contribution of source (invasion probability):
    q.year[y] <- mean(q[start[y]:end[y]])
    # proportion new arrivals:
    arrival.year[y] <- mean(new.arrival[start[y]:end[y]])
    # proportion invading:
    invade.year[y] <- mean(invading[start[y]:end[y]])
    # proportion established
    established.year[y] <- mean(established[start[y]:end[y]])
    # proportion NOT established (i.e., not "native" to Portland):
    Not.established.year[y] <- 1- established.year[y]
    # average time since arrival for each year:
    Time.year[y] <- mean(Time[start[y]:end[y]])
```

```
        # Keep track of which cities the beetles came from in each year:
        for(c in 1:Ncities){
           Source.City.year[c,y] <- mean(Source.City[c,start[y]:end[y]])
           }
    }

        # Keep track of which cities the beetles came from overall:
        for(c in 1:Ncities){
           Source.City.ave[c] <- mean(Source.City[c,])
           }

}
```
